# Editorial: Community Health Workers Practice From Recruitment to Integration

**DOI:** 10.3389/fpubh.2022.841081

**Published:** 2022-02-17

**Authors:** Julie Ann St. John, Durrell J. Fox, E. Lee Rosenthal, Lily K. Lee

**Affiliations:** ^1^Department of Public Health, Texas Tech University Health Sciences Center, Graduate School of Biomedical Sciences, Lubbock, TX, United States; ^2^Health Services Department, JSI Research & Training Institute, Atlanta, GA, United States; ^3^Texas Tech University Health Sciences Center El Paso, El Paso, TX, United States; ^4^San Manuel Gateway College, Loma Linda University Health, Loma Linda, CA, United States

**Keywords:** Community Health Workers (CHWs), integration, recruitment, training, sustainability

At no time like the present has the role of Community Health Workers (CHWs) received so much attention given their direct capacity to address community-centered health. The COVID-19 pandemic and the unprecedented attention to the gaps in health equity, especially racial and social justice issues, has highlighted CHWs' contributions to improve overall population health outcomes (1). However, this spotlight on CHWs as a solution to challenges has occurred previously. For example, CHWs were actively involved in reducing U.S. infant mortality through initiatives like Healthy Start in the early 1990s; further, CHWs actively assisted communities through disasters like Hurricane Katrina in 2005. Additionally, the Patient Protection and Affordable Care Act (2010) identified CHWs in several provisions, including in the Public Health and Prevention Fund and the National Health Care Workforce Commission. A growing number of state Medicaid programs are working on integration of CHWs into their reimbursable services to address social determinants of health. States continue to pass policies that support CHWs—including optional and required certification programs. On the surface, CHWs appear involved and integrated but is that the case? Are CHWs fully engaged in equitable ways in CHW recruitment, training, and integration?

Today CHWs and their allies participate in meetings with other members of the public health and healthcare fields talking about this novel CHW intervention, yet CHWs are often not even identified as part of inter-professional teams. Numerous organizations, let alone some community members, do not understand the presence, roles, and power of CHWs on the frontlines of public health domestically and internationally. In light of the U.S.'s current substantial investment in CHWs during the COVID-19 pandemic, we must ask if CHWs programs will be the first to be downsized as funds dissipate. Despite significant investments, CHW jobs are often part-time, without benefits, and without sufficient workspaces and equipment (such as desks and computers). We underinvest in CHWs and even isolate them so that they are not fully integrated into the fabric of our health and human services systems. Yet, CHWs can weave us into whole cloth and fill many gaps.

As an editorial team, we call for supporting the development of CHW peer reviewers, which will require time and management of hierarchies intended to support an academic expert practice perspective. We further acknowledge the need for Institutional Review Board reviewed research but also encourage more publication of the documentation and evaluation of daily practice of field-based CHW public health programming.

In this e-book special issue, we called for CHW models focused on recruitment, training, and integration strategies. We invited CHWs as peer reviewers of abstract proposals ultimately featured in this issue.

## Recruitment

Successful CHW-integrated programs begin with the right CHW recruitment.

The literature and as well as this special issue lack attention to recruitment. One of fourteen articles specifically addressed CHW recruitment. McCarthy et al. outline a recruitment and selection process for a *navegante* training program. St. John et al. discuss CHW psychosocial determinants, which could/should potentially influence CHW recruitment. This issue further demonstrates the need for the right/proper recruitment, which became more evident throughout the COVID-19 pandemic. The National Association of Community Health Workers (NACHW) policy platform calls on “Public and Private Institutions to Respect, Protect, and Partner with Community Health Workers to Ensure Equity During the Pandemic and Beyond” (n.d.).

## Training

This issue provides a wide array of CHW training program models. For example, George et al. describe a conceptual approach for CHW curriculum development focused on clinical settings while Lee et al. discuss training methods and deliveries to strengthen CHW workforce readiness capacities in clinical and practice settings. Rajabiun et al. highlight a CHW training program (HIV/chronic disease focused) that engages the National CHW Core Consensus (C3) project framework (2). Elkugia et al. describe strengths of a scalable CHW-led home visit training program: community based participatory approach, continuous engagement, RE-AIM evaluation framework, and continuous adaption through integrating lessons learned—Byrd-Williams et al. summarize key findings from a CHW survey during COVID-19 and conclude with recommendations related to training needs for responding to and serving during public health emergencies. St. John et al. report on CHW psychosocial determinants and implications of findings for CHW training and interventions. Lastly, Zheng et al. summarize findings from an online CHW training program for high school students—emphasizing instructional design processes. More recently—as evident in a number of articles in this issue—thanks to the work of the C3 project, NACHW, APHA (American Public Health Association), and a number of state-level efforts, there has been growing consensus on core standards and best practices for CHW trainings evident in this issue: proper evaluation, consent, and CHW inclusion. Yet, training programs still vary in scope and implementation of core training standards—highlighting an ongoing gap in CHW training and implementation.

## Integration

Several CHW integration models appear in the literature—ranging from community outreach and engagement to CHWs integration into complex, multidisciplinary clinical teams. This collection provides additional integration models and recommendations. The case study by Paulson et al. summarize findings from an evaluation of the integration of community health advocates (CHAs) into the care team, concluding with integration best practices and barriers. Sabo, O'Meara et al. provide recommendations for strengthening the Community Health Representative (CHR) workforce and increasing integration of CHRs within teams; similarly, Aminawung et al. recap integration strategies of formerly incarcerated CHWs into primary care teams. Sabo, Wexler et al. share findings from an assessment of organizational capacity for system change and CHW integration, while Barbero et al. utilize their conceptual model to evaluate implementation of statewide CHW workforce development opportunities. While building this literature on CHW integration through lessons learned on organizational readiness to fully and equitably integrate CHWs, we observed that gaps in integration methods persist.

In short, these 14 articles further provide evidence of CHW recruitment, training, and integration best practices and point to future areas of need. As manuscripts were submitted and reviewed, we identified areas for further exploration and publication. Within the CHW recruitment area, we should expand the availability of published research focused on effective strategies to recruit and retain CHWs from the communities served. Articles on CHW recruitment readiness would particularly assist the numerous agencies looking to hire CHWs for the first time. Implementing and documenting effective strategies to develop and sustain accessible CHW core and continual training available to CHWs in all states will benefit the future of the CHW field given that access to CHW core and ongoing training impacts the effectiveness and sustainability of the CHW workforce. Equitable CHW integration into health, public health, community wellness, and health equity teams need additional exploration and more data and publications that examine CHW integration on macro and micro levels to assist in guiding continued efforts to integrate CHWs as valued and respected members across all health and public health teams.

As stated previously, there is unprecedented attention and investment in CHWs, and the future of the field depends upon ensuring the elevation of CHWs through evaluation and research related to CHWs' roles in assisting the U.S. to achieve health equity through addressing social determinants of health; disparities; responding to past, current, and future pandemics; and other health and environmental disasters. While COVID-19 brought death and despair, the pandemic also exposed persistent disparities and inequities in the U.S. Similar to the HIV pandemic, COVID-19 provided an opportunity for CHWs to further demonstrate our value and effectiveness in addressing disparities and inequities. The pandemic and social justice movement has led to unprecedented investments in CHWs and supporting CHWs' roles in addressing social determinants of health, increasing access to accurate information and health education, and in expanding access to care.

Our editorial team included one long-serving CHW and three long-time CHW allies, all with decades of experience serving diverse communities across the U.S. Our team assembled in the spirit of CHW self-determination and the mantra of “nothing about us, without us.” We engaged CHWs and CHWs allies in every aspect—from developing the call for abstracts and manuscripts to the review processes. Putting the “peer” in “peer review” journal and publishing manuscripts that included CHWs as part of the author teams were two of the aims of our editorial team; we encouraged author teams to include CHWs and happily report that a number of manuscripts achieved this goal.

This e-book sheds light on strategies related to effective CHW recruitment, training, and integration. Our editorial team recognizes these foundational components of CHW workforce development need continuous support and exploration due to the impact and implications of these components for sustainability of the field. We hope findings shared in these articles inform your work and inspire CHWs and allies alike to help to fill the gaps revealed by these talented practitioners and researchers.

## Author Contributions

JS, DF, ER, and LL all contributed to the writing and review of this editorial. All authors contributed to the article and approved the submitted version.

## Conflict of Interest

The authors declare that the research was conducted in the absence of any commercial or financial relationships that could be construed as a potential conflict of interest.

## Publisher's Note

All claims expressed in this article are solely those of the authors and do not necessarily represent those of their affiliated organizations, or those of the publisher, the editors and the reviewers. Any product that may be evaluated in this article, or claim that may be made by its manufacturer, is not guaranteed or endorsed by the publisher.
